# Vasoactive intestinal polypeptide plasma levels associated with affective symptoms and brain structure and function in healthy females

**DOI:** 10.1038/s41598-020-80873-2

**Published:** 2021-01-14

**Authors:** Rozalyn A. Simon, Nawroz Barazanji, Michael P. Jones, Olga Bednarska, Adriane Icenhour, Maria Engström, J. Paul Hamilton, Åsa V. Keita, Susanna Walter

**Affiliations:** 1 Center for Medical Image Science and Visualization (CMIV), Linköping University, Linköping, Sweden; 2 Division of Diagnostics and Specialist Medicine, Department of Health, Medicine, and Caring Sciences, Linköping University, Linköping, Sweden; 3 Division of Inflammation and Infection, Department of Biomedical and Clinical Sciences, Linköping University, Linköping, Sweden; 4 Department of Psychology, Macquarie University, Sydney, Australia; 5 Institute of Medical Psychology and Behavioral Immunobiology, University Hospital Essen, University of Duisburg-Essen, Essen, Germany; 6 Department of Biomedical and Clinical Sciences, Center for Social and Affective Neuroscience, Linköping University, Linköping, Sweden; 7 Division of Surgery, Orthopedics and Oncology, Department of Biomedical and Clinical Sciences, Linköping University, Linköping, Sweden

**Keywords:** Neuroscience, Psychology, Signs and symptoms

## Abstract

Vasoactive intestinal polypeptide (VIP) is a neuroendocrine peptide distributed throughout the human body, including the CNS, where it is particularly abundant in brain regions associated with anxiety and depression. Based on earlier studies indicating that peripheral VIP may cross through the blood–brain barrier, we hypothesized plasma VIP levels to be associated with symptoms of anxiety and depression, as well as brain volume and resting-state functional connectivity in the amygdala, hippocampus, parahippocampus, and orbitofrontal cortex. Plasma VIP concentrations and anxiety/depression symptoms were measured in 37 healthy females. Functional and structural magnetic resonance imaging were used to evaluate functional connectivity and brain volume respectively, and their associations with VIP concentrations within brain regions associated with anxiety and depression. Negative correlations were found between VIP levels and symptoms of anxiety (*r* = − 0.44, *p* = 0.002) and depression (*r* = − 0.50, *p* = 0.001). Functional connectivity demonstrated significant VIP-dependent positive associations between the amygdala seed region with both the right parahippocampus (*t*_(33)_ = 3.1*, p*_*FDR*_ = 0.02) and right lateral orbitofrontal cortex (OFC; *t*_(33)_ = 2.9*, p*_*FDR*_ = 0.02). Moreover, VIP concentrations were significantly, positively correlated with brain volume in the left amygdala (*r* = 0.28, *p* = 0.007) and left lateral OFC (*r* = 0.29, *p* = 0.004). The present findings highlight a potential role for VIP in the neurobiology of affective symptoms.

## Introduction

Vasoactive intestinal polypeptide (VIP), first isolated and identified from porcine intestines, is a neuropeptide hormone found throughout the peripheral and the central nervous system (CNS)^1–3^ with a variety of proposed functions^4–8^ which include neuroprotection through the inhibition of proinflammatory mediators^9–11^. The first studies to investigate VIP distribution in the human brain reported high concentrations in the amygdala, hippocampus, and prefrontal regions^9,12^, all of which are associated with anxiety^13–17^, depression^18^, and emotional learning^19–21^ including fear conditioning^22–25^. Subsequent evidence from stress studies conducted in animals indicates that VIP plays a role in modulating learning and memory mechanisms in both the prefrontal cortex and the hippocampus via neuronal relays to the amygdala^26–29^. More recent evidence in mouse models indicates that during adverse events, VIP interneurons in the amygdala inhibit inhibitory neurons, thereby contributing to the disinhibition of excitatory projection neurons, allowing for memory formation, discrimination between important or irrelevant information, and adaptive response during unpredicted and adverse events^30,31^. In addition, Krabbe et al. showed that VIP-expressing interneurons have monosynaptic connections from the amygdala, a central hub of the emotion processing network, to the hippocampus, parahippocampus, and orbitofrontal cortex (OFC), among other regions, establishing the structural connections linking these anatomical regions in relation to VIP^30,31^.

Although VIP has been studied in animal models in relation to anxiety^26^, depression^32^, fear conditioning^33^, and memory^6,34^, VIP research in humans is rather limited. One human study providing evidence for a link between VIP and psychological measures was conducted in individuals with major depressive disorder which reported that VIP in both sweat and plasma samples negatively correlated with anxiety and depression scores^35^. A number of magnetic resonance imaging (MRI) studies in humans have linked anxiety^36^ and depression^18,37^ to abnormal structure and function within VIP-rich areas related to emotion processing and modulation but none in direct relation to VIP. For example, amygdala volume was found to be negatively associated with trait anxiety in healthy subjects^38^, and reduced structural integrity of white matter tracts between the amygdala and prefrontal regions was shown to be predictive of trait anxiety^39^, while disrupted resting-state functional connectivity between the amygdala and the OFC was seen in anxiety disorders^40–43^. In one therapeutic study, patients with chronic inflammatory response syndrome received intranasal treatment with VIP which was later associated with an increase in the grey matter volume of the amygdala^44^, yet little remains known about VIP and brain volume in healthy individuals. Although these findings in humans point toward a potential association between affective disorders and VIP neuropeptides, little is understood about VIP’s specific relation to central processing in regions of emotion, such as the amygdala, in healthy individuals. Since there is evidence that VIP can penetrate the blood–brain barrier^45^ we explored potential associations between VIP levels in blood plasma and CNS-related measures in healthy individuals. Our aims were to determine if VIP concentrations in peripheral blood plasma were negatively associated with symptoms of anxiety and depression in healthy individuals and to investigate potential relationships between plasma VIP and CNS measures of grey matter volume and resting-state functional connectivity within specific VIP-rich regions of emotion processing, namely the amygdala, hippocampus, parahippocampus, and orbitofrontal cortices.

## Materials and methods

### Subjects

37 healthy female participants with a mean age of 34 years (range 20–55 years) and a mean BMI of 23.6 kg/m^2^ (range 17.6–34.1 kg/m^2^) were recruited by advertisement from the University Hospital, Linköping, Sweden. As abnormal VIP levels have been associated with gastrointestinal disorders^46–48^, individuals with a medical history of gastrointestinal symptoms or complaints were excluded to avoid potential confounds. Since VIP is known to be involved in the control of satiety feeding behavior, body mass index (BMI) was measured^1^. Exclusion criteria were further established via interviews with participants to verify they did not suffer from any organic gastrointestinal disease, allergy, metabolic or neurological disorders, and both past and current severe psychiatric disease (e.g., schizophrenia, bipolar disorder, etc.). All participants were required to be fluent in Swedish. The Regional ethical review board in Linköping approved the study (Dnrs. 2013/506-32; 2014/264-32) and all subjects gave their written informed consent. All experiments were performed in accordance with relevant guidelines and regulations.

### Questionnaires

#### Hospital Anxiety and Depression Scale (HADS)

HADS was used to estimate symptoms of depression and anxiety^49,50^. The scale consists of seven items for depression (HADS-D) and anxiety subscales (HADS-A), respectively, with scores on each subscale ranging from 0 to 21. Cut-off values are indicated as ≥ 8 for mild symptoms of anxiety or depression and ≥ 11 as clinically significant for both the HADS-D and HADS-A.

### Quantification of VIP in plasma by enzyme immunoassay (EIA)

Fasting venous blood samples were collected between 7:30 a.m. to 5 p.m. within a mean of 6 days (1–14) of the MR imaging and questionnaire administration. The participants were instructed to refrain from the use of anti-inflammatory drugs within 24 h prior to blood draw. No systematic differences emerged between groups with respect to the time blood draw (data not shown). Blood samples were collected in EDTA-treated tubes and a mixture of 1.3 mg EDTA and 50 μl Trasylol 10 000 KiE was added to each ml of blood. After centrifugation, 3400*g* for 15 min in 4 °C, plasma was redrawn and frozen in − 80 °C until analysed using a VIP-enzyme immunoassay kit (Phoenix Pharmaceuticals, Germany). Upon analysis, undiluted plasma, standard samples, and positive/negative controls were added to pre-coated plates in duplicates and further analyzed according to manufacturer’s instructions. Absorbance was measured at 450 nm in VERSAmax Tunable Microplate Reader (Molecular Devices, CA, USA). By using Softmax pro 5 (Molecular Devices), a standard curve based on the standard samples was generated, from which the plasma concentrations of VIP were calculated.

### Magnetic resonance imaging

#### fMRI acquisition

All MR images were acquired using a 32-channel head coil on a 3 T Philips Ingenia MRI scanner (Philips Healthcare, Best, The Netherlands) at the Center for Medical Image Science and Visualization at Linköping University, Sweden. Ten minutes of eyes-closed, resting-state fMRI data were acquired with a single-shot, gradient-echo EPI sequence with repetition time and echo time (TR/TE) = 2000/37 ms; voxel size = 3.59 × 3.59 × 4 mm^3^; 28 slices; SENSE factor = 2) that covered the whole brain. T1-weighted 3D FFE images were acquired in all participants using the following parameters: inversion preparation and delay 900 ms, SAG-plane, FOV 256 × 240 × 170 mm^3^, resolution 1 × 1 × 1 mm^3^, flip angle 9°, TR = 7 ms, TE = 3.2 ms, TA = 5:34 min.

#### Resting-state fMRI analysis

**Figure 1:**
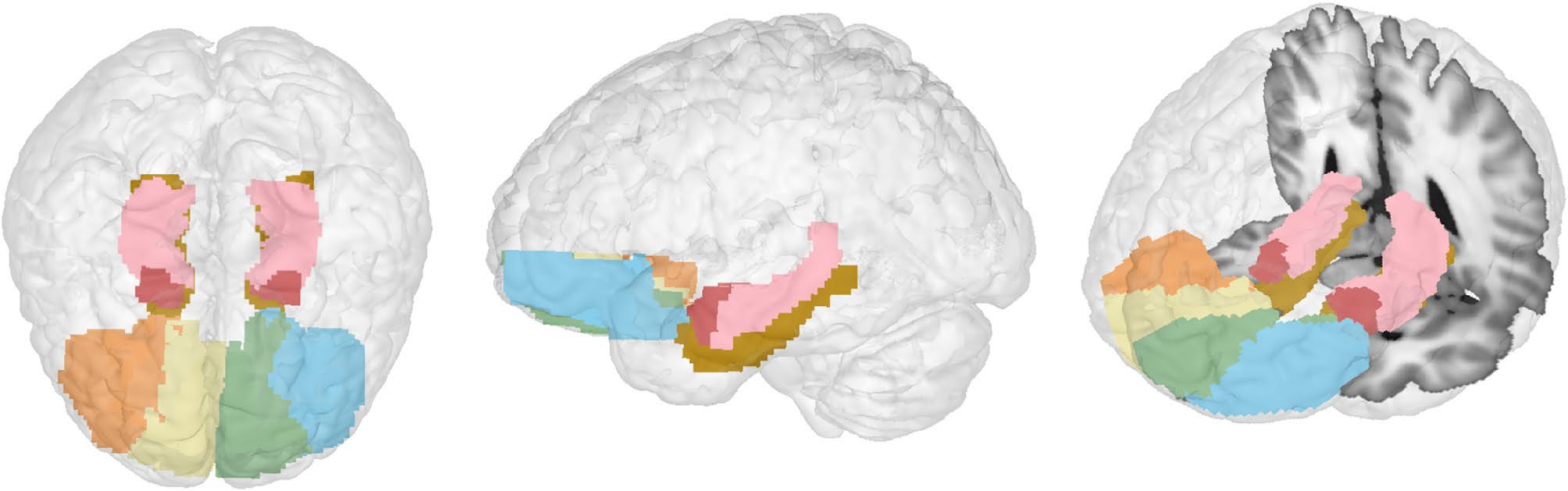
Anatomical regions of interest used in the resting-state functional connectivity and grey matter volume analyses. Left: Top view of all regions of interest. Middle: Left side view. Right: Left side view tilted with slice for reference. Dark pink-bilateral amygdala, orange-right lateral OFC, yellow-right medial OFC, green-left medial OFC, blue-left lateral OFC, light pink-hippocampus, brown-parahippocampal gyrus.

Resting-state fMRI data were preprocessed and analyzed using the CONN functional connectivity toolbox^51^ (ver. 18a) (http://www.nitrc.org/projects/conn) in conjunction with SPM 12 (Wellcome Dept. of Cognitive Neurology. London, UK; http://www.fil.ion.ucl.ac.uk/spm). Preprocessing was done using the standard pipeline in CONN with realignment and unwarping for estimation and correction of subject motion; slice timing correction, ART-based outlier detection and scrubbing for the removal of mean signal intensity and motion artefacts, segmentation and normalization of the functional images to Montreal Neurological Institute (MNI) coordinates, and smoothing (8 mm full width half maximum (FWHM) Gaussian kernel). To denoise the functional images, a band-pass filter of 0.008–0.09 Hz with linear detrending was used. Functional connectivity between regions of interest (ROIs) was calculated using ROI-to-ROI analysis. Automated anatomical labelling (aal) atlas^52^ ROIs included bilateral amygdala, hippocampus, parahippocampal gyri, and lateral and medial OFC regions (Fig. 1). Figures 3 and 4a were generated using CONN software.

ROI-to-ROI analyses were performed with seeds in bilateral amygdala and targets comprising the bilateral hippocampus, parahippocampal gyri, and lateral and medial OFC regions *(false discovery rate (FDR) corrected p*_*FDR*_ < 0.05).

#### Regional grey matter probability

Grey matter volume (GMV) measurement was performed on the T1 weighted images using CAT12 toolbox (CAT, http://dbm.neuro.uni-jena.de/vbm/) in SPM running on MATLAB (R2017a, MathWorks Natick, Massachusetts, USA)^53^. Each T1 image was reoriented so that all the images would have the anterior commissure as the point of origin. Prior to segmentation, a non-linear deformation field for each image was estimated. Using a tissue probability map, each image was segmented into grey matter, white matter, and cerebrospinal fluid and spatially normalized into MNI space. For between-subject registration and grey matter modulation, diffeomorphic anatomical registration using exponentiated lie algebra (DARTEL) toolbox was used^54^. Final modulation of the voxel values was done according to the Jacobian determinant of the deformation field initially estimated. Using the same aal atlas ROIs as in the fMRI analysis, GMP was extracted using the MANGO image processing system (Research Imaging Center, UTHSCSA; http://ric.uthscsa.edu/mango). Using the ROI statistics in MANGO we extracted the grey matter probability for each individual in each ROI. Figure 1 was generated in MANGO.

### Statistical analysis

For functional connectivity, we compared two methods of analysis, first, a nonparametric multiple regression was conducted in CONN functional connectivity toolbox^51^ (ver. 18a) (http://www.nitrc.org/projects/conn) in conjunction with SPM 12 (Wellcome Dept. of Cognitive Neurology. London, UK; http://www.fil.ion.ucl.ac.uk/spm) using the mean connectivity values with VIP as the predictor while controlling for both HADS anxiety and depression scores due to previously published results showing that both structure and function of the amygdala can be associated with anxiety and depression^42,55,56^. For comparison, we also conducted a multiple linear regression which employed a nonparametric bootstrap with 2000 iterations in which the association was adjusted for anxiety and depression due to violation of assumptions of the regression t-test for confounding variables. When evaluating the association between VIP and brain volume measures, this same multiple linear regression with nonparametric bootstrap was used in which the association was adjusted for age, anxiety, depression, and total intracranial volume. The adjusted association between VIP and either volume or connectivity measures was displayed graphically through partial regression plots generated from the bootstrapping approach. In addition, we conducted Mann–Whitney between-groups comparison of bilateral regional volumes with a Bonferroni–Dunn correction. Graphs and t-tests performed in Graphpad Prism version 8.0.0 for Windows, GraphPad Software, San Diego, California, USA, www.graphpad.com.

## Results

**Figure 2:**
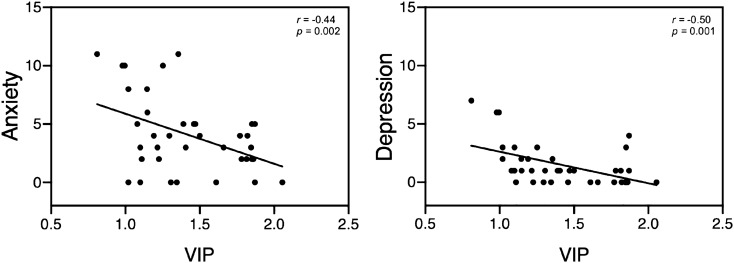
VIP Pearson correlations with Hospital anxiety and depression scale (HADS), p-values derived from non-parametric bootstrapping. *VIP *vasoactive intestinal polypeptide.

Demographic, blood, and clinical data for participants are shown in Table 1. There was a significant negative association between VIP and anxiety (*r* = − 0.44, p = 0.002) and depression (*r* = − 0.50, p = 0.001), respectively (Fig. 2). BMI was not significantly correlated with VIP or HADS scores.

**Table 1:** Characterization of healthy participants.

Participant data (N = 37)	Mean (range)
Age	33.51 (20–55)
Body mass index, kg/m^2^	23.60 (17.6–34.1)
Plasma-VIP concentrations	1.42 (0.81–2.06)
HAD anxiety	4.08 (0–11)
HAD depression	1.49 (0–7)

### Resting-state connectivity

**Figure 3:**
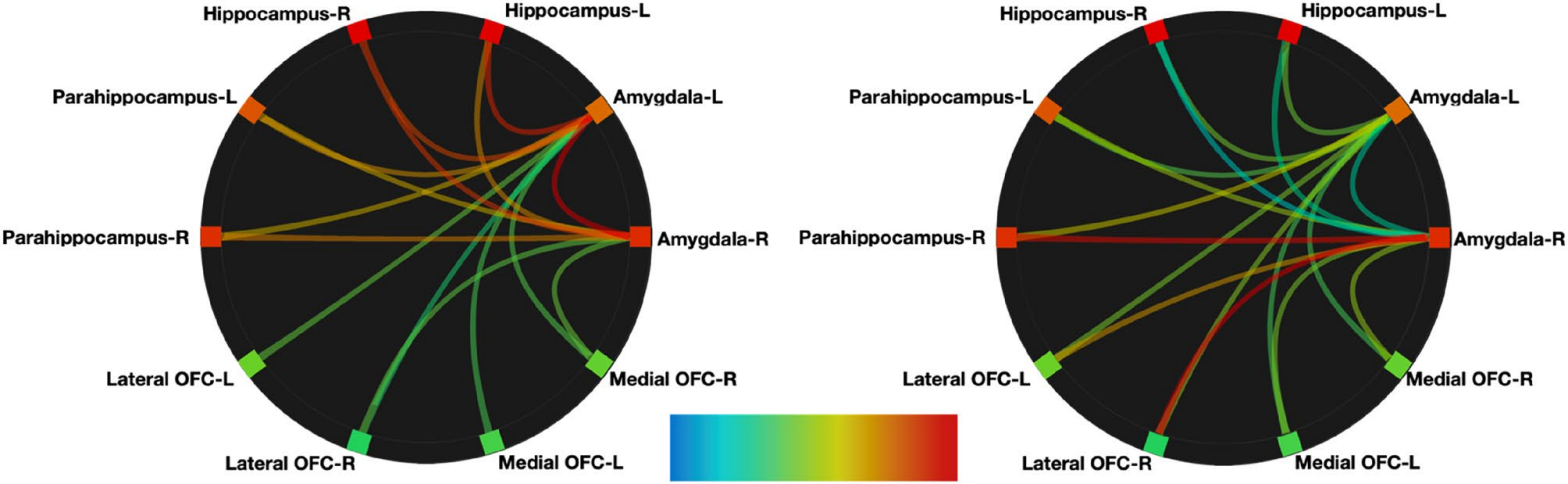
Connectome rings showing relative intrinsic connectivity between the anatomical ROIs of the bilateral amygdala to bilateral hippocampus and parahippocampal gyri and the left lateral and right medial OFC-orbitofrontal cortices. Color and thickness of path indicates strength of the connectivity with red being the strongest and blue weakest (unthresholded). Left: VIP-independent intrinsic connectivity. Right: VIP-associated connectivity controlling for anxiety and depression.

ROI-to-ROI analyses of resting-state data were performed to examine resting-state functional connectivity within regions of emotion processing, with seeds in bilateral amygdala and targets comprising the bilateral hippocampus, parahippocampal gyri, and lateral and medial OFC regions. We first determined the relative intrinsic VIP-independent strength of connectivity between the selected regions of the network in our participant group. We found that both the left and right amygdala had intrinsic functional connectivity to all regions in a relatively anatomic distance-dependent manner (unthresholded) (Fig. 3, left).

**Figure 4:**
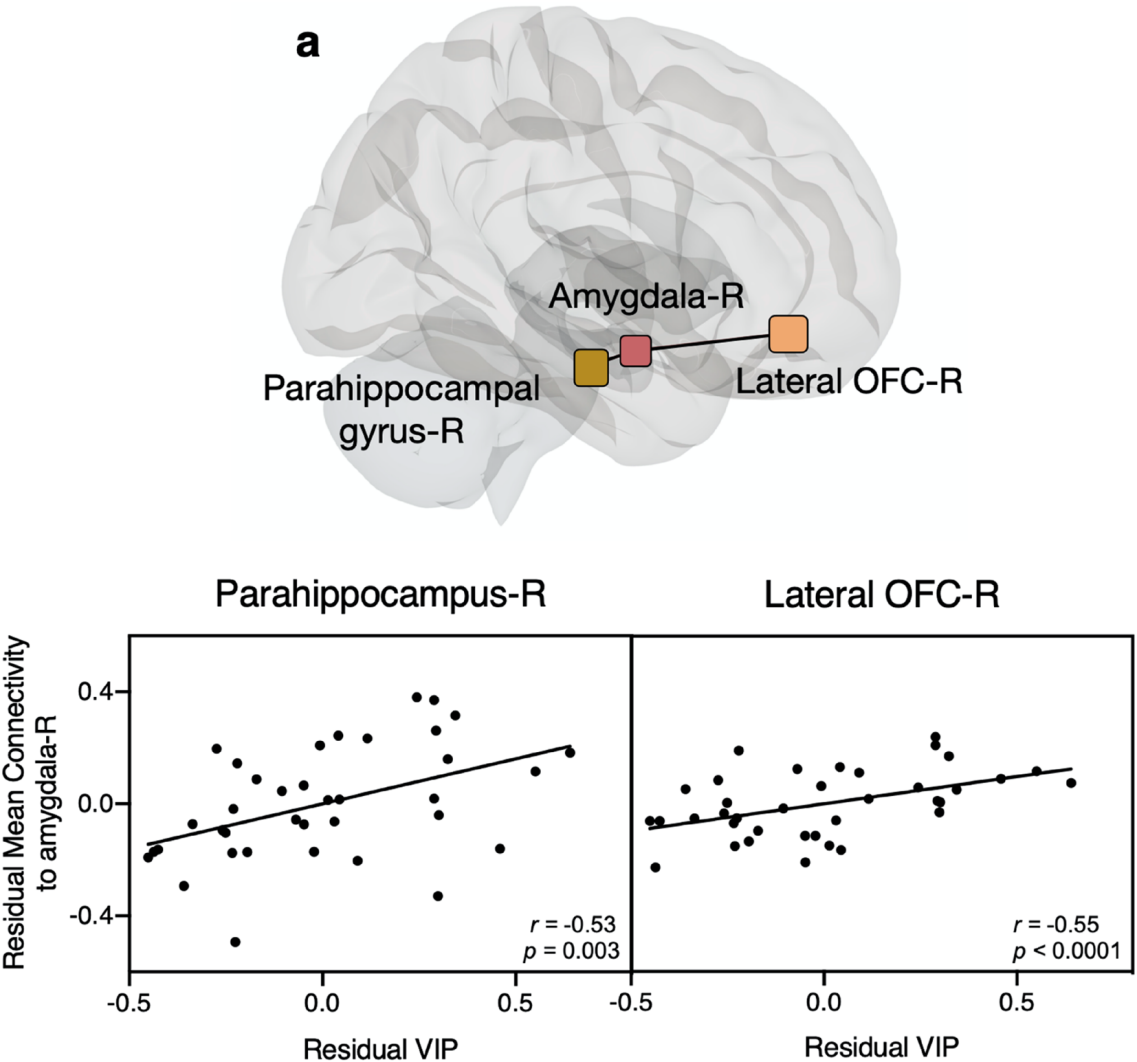
Significant VIP-associated connectivity in resting-state functional MRI. (**a**) Visual representation of regions with significant VIP-associated connectivity within the right hemisphere (*p*_*FDR*_ < 0.02). (**b**) Partial regression plots from bootstrapping showing the relationship between adjusted mean connectivity values and residual VIP values between the right amygdala and either the right parahippocampal gyrus or the right lateral OFC (Pearson’s *r* and adjusted *p*-values from bootstrapping). *OFC *orbitofrontal cortex, *VIP *vasoactive intestinal polypeptide, *R *right.

Second, we investigated the relative intrinsic VIP-associated strength of connectivity between the selected regions of the network in our participant group while controlling for anxiety and depression (unthresholded) (Fig. 3, right). Then, to investigate if plasma levels of VIP were associated with functional connectivity in the selected regions, we performed a multiple regression analysis between VIP and functional connectivity values between bilateral amygdala seed regions and all target regions including the hippocampus, parahippocampal gyri, and lateral and medial OFC regions while controlling for both HADS anxiety and depression. Resting-state connectivity which correlated with VIP plasma levels was found between the right amygdala and both the right parahippocampus (*t*_(33)_ = 3.1*, p*_*FDR*_ = 0.02) and right lateral OFC (*t*_(33)_ = 2.9,* p*_*FDR*_ = 0.02) (Fig. 4). No significant VIP-associated connectivity was found between the right amygdala and remaining bilateral regions nor between the left amygdala in connectivity with any bilateral regions (see Supplementary Table 1). We additionally tested age, anxiety, depression, and total intercranial volume independently as variables of interest but found no significant effect of any of these independent variables on connectivity within these regions of interest. Results from regression using nonparametric bootstrapping gave similar results (Fig. 4).

### Grey matter volume

Regional GMV analysis was conducted for the bilateral amygdala, hippocampus, parahippocampus, left and right medial and lateral OFC (Table 2). Results from the regression analysis with bootstrapping controlling for age, anxiety, depression, and total intracranial volume, showed VIP concentrations significantly positively correlated with brain volume in the left amygdala (*r* = 0.28, *p* = 0.007) and left lateral OFC (*r* = 0.29, *p* = 0.004). In addition, between-groups Mann–Whitney t-tests showed the left amygdala, hippocampus, and lateral OFC to be significantly larger than right-sided counterparts, while the right parahippocampus was significantly larger than the left, with no significant difference between medial OFC regions (Supplementary Table 2).

**Table 2:** Gray matter volumes, reported as proportion of gray matter within each ROI.

Brain region	Means (variance)
L amgydala	0.69 (0.003)
R amygdala	0.58 (0.003)
L hippocampus	0.54 (0.002)
R hippocampus	0.49 (0.002)
L parahippocampus	0.46 (0.002)
R parahippocampus	0.51 (0.002)
L medial orbitofrontal cortex	0.47 (0.003)
R medial orbitofrontal cortex	0.45 (0.003)
L lateral orbitofrontal cortex	0.46 (0.002)
R lateral orbitofrontal cortex	0.39 (0.001)

## Discussion

VIP is a neuroendocrine peptide distributed throughout the human body, including the central nervous system (CNS), where it is particularly abundant in brain regions associated with emotional processing, specifically anxiety and depression. Our interest herein was to investigate central processing associated with VIP in healthy females through potential associations with symptoms of anxiety and depression, as well as brain structure and function within anatomically connected regions involved in emotional processing.

Extending previous reports in a patient group with major depression in remission^35^, we observed VIP plasma levels to be negatively associated with overall low symptoms of anxiety and depression in healthy individuals. Based on previous evidence suggesting that VIP crosses the BBB unidirectionally, and centrally secreted VIP is restricted to the CNS^57^, these findings together suggest a link between peripheral neuropeptide concentrations and psychological symptoms in both clinical and non-clinical populations.

In terms of our functional connectivity analysis, we chose the amygdala as the seed for our analysis as it is a central hub for emotional processing in the brain. In addition to the amygdala, central processing associated with fear and anxiety, has also been shown to involve other VIP-rich regions which are connected to the amygdala by interneurons—such as the hippocampus and prefrontal cortices^9,12,22^ thus forming an anatomically connected “microcircuit”^31^. VIP has also recently been described to have a “feed-forward” disinhibitory effect on excitatory projection neurons within this microcircuit^58,59^. VIP’s indirect disinhibition of excitatory projection neurons is proposed to allow for memory formation and discrimination between important and irrelevant information thereby modulating conditioned responses during unpredicted and adverse events^30,31^. As functional connectivity represents a proxy measure for synchronized neuronal activity, the observed positive association between VIP and functional connectivity between these interneuron-coupled regions of the emotional processing network during rest, could indicate that VIP’s disinhibitory effect within this network extends into intrinsic brain function, and is not limited to adverse events. The observation that there was no significant anxiety or depression-related connectivity within these emotion-associated regions may be due to the fact that HADS scores in this group of healthy female participants were subclinical.

**Figure 5:**
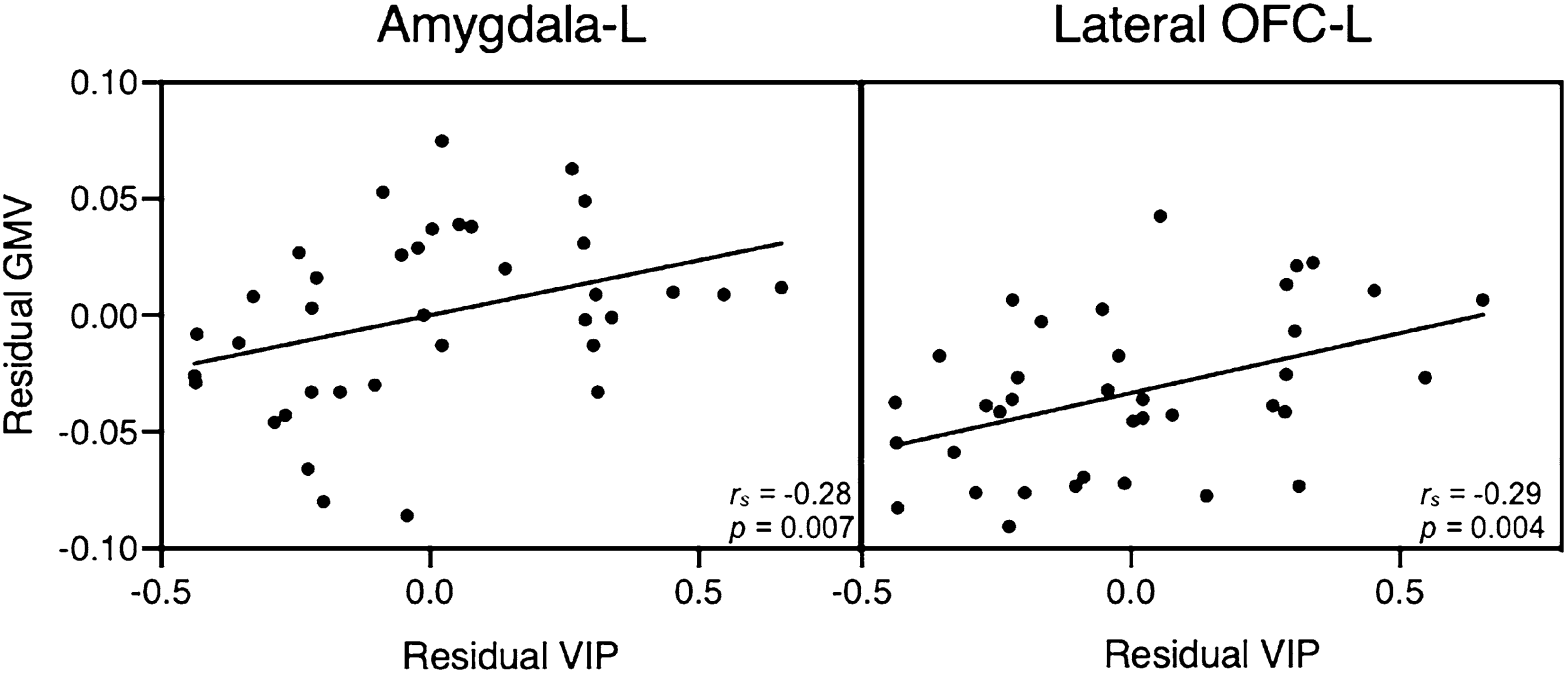
Partial correlation between VIP and grey matter. The graphs show the partial regression plot of the adjusted left amygdala grey matter volume and residual VIP (left) and the adjusted left lateral orbitofrontal cortex grey matter volume and residual VIP (right) influenced by the covariates age, total intracranial volume, HADS anxiety and depression.

Complementing and extending our functional findings, we further provide first evidence of a positive association between VIP and grey matter volume in the left amygdala and left lateral OFC in healthy female participants. To our knowledge this has not been reported before, though Shoemaker et al. did report a volumetric increase in several brain regions, among them the hippocampus and amygdala, after administering intranasal VIP to patients with chronic inflammatory response syndrome^44^. VIP has previously been described as a neuroprotective transmitter through several suggested mechanisms such as neuronal differentiation by activity-dependent neurotrophic factor released upon astroglia cell activation^60^. Korkmaz et al. showed that transgenic mice models of Alzheimer’s disease receiving VIP treatment, had less plaque accumulation and less cortical atrophy compared to those receiving saline^61^. In light of these and other VIP studies, our results showing a weak yet significant, positive association between VIP and grey matter volume in VIP-receptor-rich regions, lend support to the growing evidence that VIP plays a neuroprotective role (Fig. 5).

Our structural and functional findings indicated a lateralized effect. The amygdala in particular has been shown to have consistent lateralization effects in both structure^62,63^ and function^64^, showing that the left amygdala is typically larger in volume and more functionally active than the right. Our findings here somewhat agree, in that the GMV in four of the five left-lateralized ROIs were larger than right lateralized counter parts, with the exception of the parahippocampus, where the right region was larger (Supplementary Table 2). To further understand the differences between the structural results and the functional results, we checked patterns of lateralization in functional connectivity. Here we found that in both the left and right amygdala respectively, the relative strength of connectivity was strongest with the right-counterpart of every bilateral target region (seen in Fig. 3, right; and Supplementary Table 1). Thereby, each amygdala respectively, was relatively more functionally connected to the right-sided target regions, compared to their left counterparts. Although the overall pattern is suggestive of a trend toward greater functional connectivity in regions of lower volume as most volumes on the right were smaller, the larger volume in the parahippocampus does not follow this trend, leaving any further speculation concerning lateralization in relation to VIP, dependent upon additional investigation.

In conclusion, we found that VIP plasma levels were positively associated with brain function and volume in regions associated with emotional processing, and inversely related to symptoms of anxiety and depression in healthy females. These structural and functional results support evidence indicating that VIP is neuroprotective^10,11^ while additionally indicating that VIP’s associations extend into some dimensions of affect. As accumulating evidence strengthens the association between depression and inflammation^10^, these findings are quite relevant considering that one of the main roles of VIP is the inhibition of proinflammatory mediators. The neuroprotective and anti-inflammatory properties of VIP are now being investigated for the treatment of neurodegenerative^65^, inflammatory, and autoimmune^66^ diseases, and for use as a biomarker in specific conditions. Our findings provide important further evidence on psychological, functional, and structural levels to support VIP as a potential target for therapeutic investigation in anxiety and depression, and in disorders of the gut-brain axis with high psychological comorbidities.

As this study was conducted with female participants, it is important to note that these results are not clearly generalizable to males. Subsequent analysis of anxiety and depression-related VIP associations would benefit from sex-specific MRI subgrouping, affective MRI tasks, the collection of a broad number of related inflammatory markers for comparison to VIP, menstrual cycle information, as well as longitudinal design in a clinically depressed patient group. In addition, differentiation between the effects of centrally and peripherally produced VIP could help to further delineate the broad number of functions currently associated with this intriguing neuropeptide.

## Supplementary Information

Supplementary Tables.

## Data Availability

The datasets generated during and/or analysed during the current study are available from the corresponding author on reasonable request.
