# Supplementary material for: Vasoactive intestinal polypeptide plasma levels associated with affective symptoms and brain structure and function in healthy females
Source: Sci Rep. 2021 Jan 14;11:1406. doi: 10.1038/s41598-020-80873-2 (PMC7809454; doi:10.1038/s41598-020-80873-2)
Supplement: Supplementary file 1 — Supplementary Tables. [file 41598_2020_80873_MOESM1_ESM.docx]

**SUPPLEMENTARY DATA**

**Supplementary table 1).** Functional results for all regions included in the connectivity analysis.

|  | **Significant difference** | **Mean rank of Left-side ROIs** | **Mean rank of Right side ROIs** | **Mean rank diff.** | **Mann-Whitney U** | **Adjusted P Value** |
| --- | --- | --- | --- | --- | --- | --- |
| **Amygdala** | Yes | 53.46 | 21.54 | 31.92 | 94 | 4.2e-10 |
| **Hippocampus** | Yes | 48.41 | 26.59 | 21.81 | 281 | 2.9e-05 |
| **Parahippocampus** | Yes | 26.36 | 48.64 | -22.27 | 272.5 | 1.8e-05 |
| **Lateral OFC** | Yes | 50.59 | 24.41 | 26.19 | 200 | 1.3e-07 |
| **Medial OFC** | No | 42.38 | 32.62 | 9.757 | 504 | 2.5e-01 |

**Supplementary table 2).** Mann-Whitney results for left and right between-group comparisons of GMV.
